# 3D printing technology combined with personalized plates for complex distal intra-articular fractures of the trimalleolar ankle

**DOI:** 10.1038/s41598-023-49515-1

**Published:** 2023-12-19

**Authors:** Hairui Liang, He Zhang, Beibei Chen, Lei Yang, Rongda Xu, Siyu Duan, Zhencun Cai

**Affiliations:** 1 Department of Orthopedics Surgery, Central Hospital Afliated to Shenyang Medical College, 5 Nanqi West Road, Shenyang, 110075 Liaoning China; 2 School of Pharmacy, Inner Mongolia Medical University, 5 Xinhua Street, Hohhot, 010107 Inner Mongolia Autonomous Region China

**Keywords:** Anatomy, Health occupations, Medical research, Signs and symptoms

## Abstract

This study investigated the effectiveness of 3D printing technology in combination with personalized custom-made steel plates in the treatment of complex distal intra-articular trimalleolar fractures, with the aim of providing a new approach to improve ankle joint function in patients. The 48 patients with complex distal intra-articular trimalleolar fractures included in the study were randomly divided into two groups: the personalized custom-made steel plate group (n = 24) and the conventional steel plate group (n = 24). A comparison was made between the two groups in terms of preoperative preparation time, hospitalization duration, surgical time, fracture reduction and internal fixation time, intraoperative fluoroscopy instances, surgical incision length, fracture healing time, follow-up duration, degree of fracture reduction, ankle joint functional recovery, and the occurrence of complications. The personalized steel plate group exhibited longer preoperative preparation time and hospitalization duration compared to the conventional steel plate group (p < 0.001). However, the personalized steel plate group demonstrated significantly shorter surgical duration, time for fracture reduction and internal fixation, reduced intraoperative fluoroscopy frequency, and a shorter overall surgical incision length (p < 0.001). Both groups displayed similar fracture healing times and follow-up durations (p > 0.05). The personalized steel plate group showed a higher rate of successful fracture reduction (87.5% vs. 79.2%, p > 0.05) and a lower incidence of complications (8.3% vs. 20.8%, p = 0.22), although these differences did not reach statistical significance. Furthermore, the personalized steel plate group exhibited superior ankle joint function scores during follow-up compared to the conventional steel plate group (p < 0.05). By utilizing 3D printing technology in conjunction with personalized custom-made steel plates, personalized treatment plans are provided for patients with complex comminuted tri-malleolar ankle fractures, enabling safer, more efficient, and satisfactory orthopedic surgeries.

## Introduction

Complex distal intra-articular fractures of the trimalleolar ankle are typically caused by high-energy injuries, presenting with significant fracture dislocation, articular surface damage, and surrounding soft tissue injuries. There is considerable individual variation, making achieving anatomical reduction of fractures and joint surfaces challenging during surgery^1–3^. Traditional plate fixation after fracture reduction may sometimes yield suboptimal results. Complex low-distal comminuted tri-malleolar fractures are considered severe intra-articular fractures, emphasizing the crucial importance of ensuring anatomical reduction and effective fixation of joint surfaces for optimal patient recovery^4^. The anatomical structure of the ankle joint is complex, and conventional imaging data often struggle to provide comprehensive information. Simultaneously, the soft tissues surrounding the ankle joint are relatively fragile, and improper handling during surgery may lead to various postoperative complications^3^.

In recent years, 3D printing technology has gradually entered clinical practice for treating complex tri-malleolar fractures. Physicians can use physical models of fractures to assess the misalignment more intuitively and accurately, aiding in intraoperative fracture reduction and reducing surgical complexity. However, current 3D printing technologies are primarily used for pre-shaping conventional plate^5,6^. If they still cannot fit effectively during surgery, further bending of the plates is required, compromising their strength and potentially causing loosening of bone fragments and joint instability. In this study, our research team utilized computer-simulated fracture reduction to customize the plates required during surgery based on the virtually restored fracture model^7–10^. The precise integration of the model with the plate enhances the accuracy of surgical planning and preoperative simulation, further reducing surgical complexity and improving the quality of surgery^11,12^. This provides a new approach for managing such fractures in clinical practice. This study aims to explore the differences in surgical outcomes in the treatment of complex low-distal comminuted tri-malleolar fractures using 3D printing technology in conjunction with personalized custom plates compared to conventional procedures.

## Materials and methods

### Patient information

A retrospective analysis was conducted on a total of 55 patients with severe distal intra-articular fractures of the trimalleolar ankle who underwent inpatient surgical treatment at the Affiliated Central Hospital of Shenyang Medical College between March 2019 and March 2022. Based on inclusion and exclusion criteria, a total of 48 fracture patients were enrolled, and these patients were randomly divided into two groups: a personalized steel plate group consisting of 24 cases and a conventional steel plate group consisting of 24 cases.

#### Inclusion criteria

① Fresh closed fractures requiring surgical intervention; ② Ankle joint fractures caused by trauma, meeting clinical fracture classification criteria, diagnosed as complex distal intra-articular fractures of the trimalleolar ankle through X-ray and CT scans (with comminuted fractures involving the medial, lateral, and posterior malleoli, encompassing the articular surfaces); ③ Age between 20 and 65 years, with mature skeletal development, and patients and their families choosing surgical treatment.

#### Exclusion criteria

① Patients with open injuries or requiring external fixation. ② Pathological fractures.③ Patients with concomitant traumatic brain injuries. ④ Patients with old fractures. ⑤ Lost-to-follow-up patients.

### Ethical approval

This study obtained approval from the Medical Ethics Committee of the Affiliated Central Hospital of Shenyang Medical College, and informed consent was obtained from patients and their families. All procedures were in accordance with the ethical standards of institutional and/or national research councils.

### Preoperative patient preparation and management

Upon admission, the affected limb is treated with plaster fixation, anti-swelling measures, pain relief, and preventive measures against lower limb deep vein thrombosis. Preoperative examinations are conducted comprehensively. Prior to surgery, all patients underwent ankle joint X-ray imaging in both anterior–posterior and lateral views (using a Philips digital radiography system from the Netherlands), as well as CT scans of the affected ankle joint with three-dimensional reconstruction (using a Philips 256-slice helical CT scanner from the Netherlands, with a slice thickness of 0.6 mm). In the personalized plate group, fracture data were virtually simulated, and 3D models were printed to customize individualized plates. Surgery was postponed until the patient's soft tissue condition improved.

### Personalized steel plate group: virtual simulation, 3D printing and customized personalized plates

**Figure 1:**
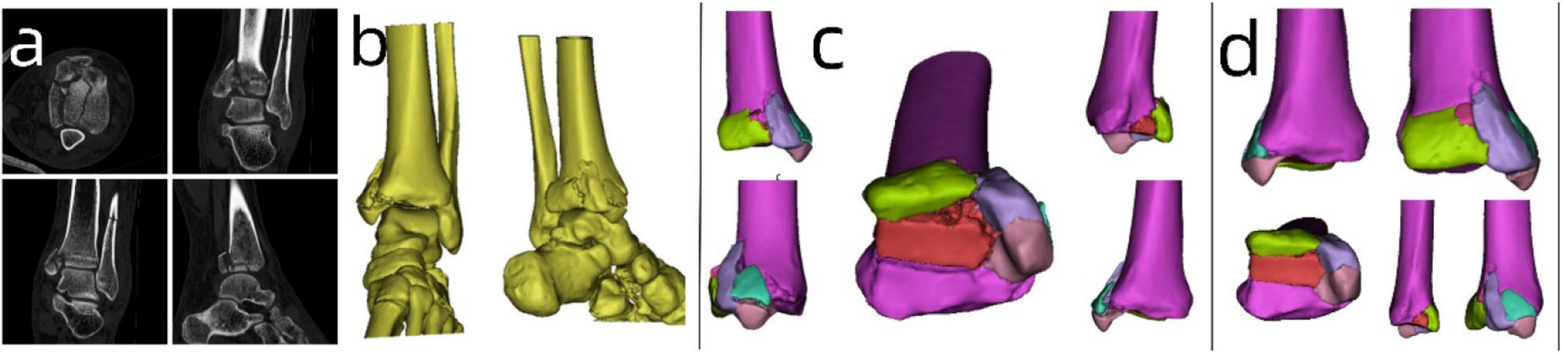
(**a**) CT imaging data of the fractured area. (**b**) Three-dimensional modeling of the fracture data using Mimics software. (**c**) Isolation of fracture fragments and color-coded annotation using the threshold circle selection function. (**d**) Virtual anatomical reduction of the fracture fragments using the move-rotate function.

**Figure 2:**
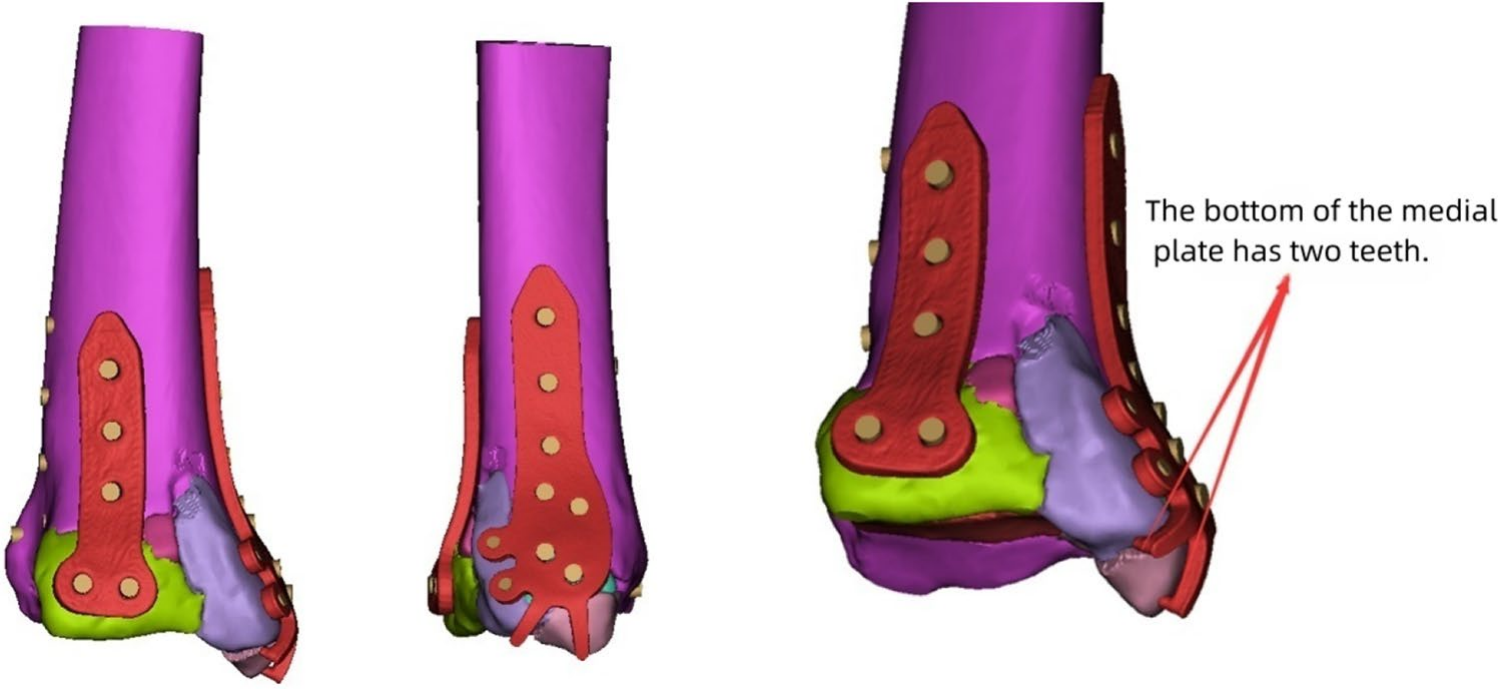
Based on the virtually reduced fracture model, a personalized customized plate scheme is tailored to determine the optimal plate placement.

The patient's CT data of the fracture site (Fig. 1a) was imported into Mimics 20.0 software (Materialise, Belgium) workstation in DICOM format. Three-dimensional modeling of the ankle joint fracture data was performed (Fig. 1b). The fracture blocks were separated and color-coded differently (Fig. 1c), followed by anatomical virtual reduction of the fracture blocks (Fig. 1d). Subsequently, the three-dimensional modeling and virtually reduced fracture data were exported in STL format and imported into FlashPrint 5 software (Flashforge, China) to print three-dimensional physical models of the fracture (Fig. 4a). Using a combination of medical and engineering expertise, a customized individualized plate design was created (Fig. 2).

**Figure 3:**
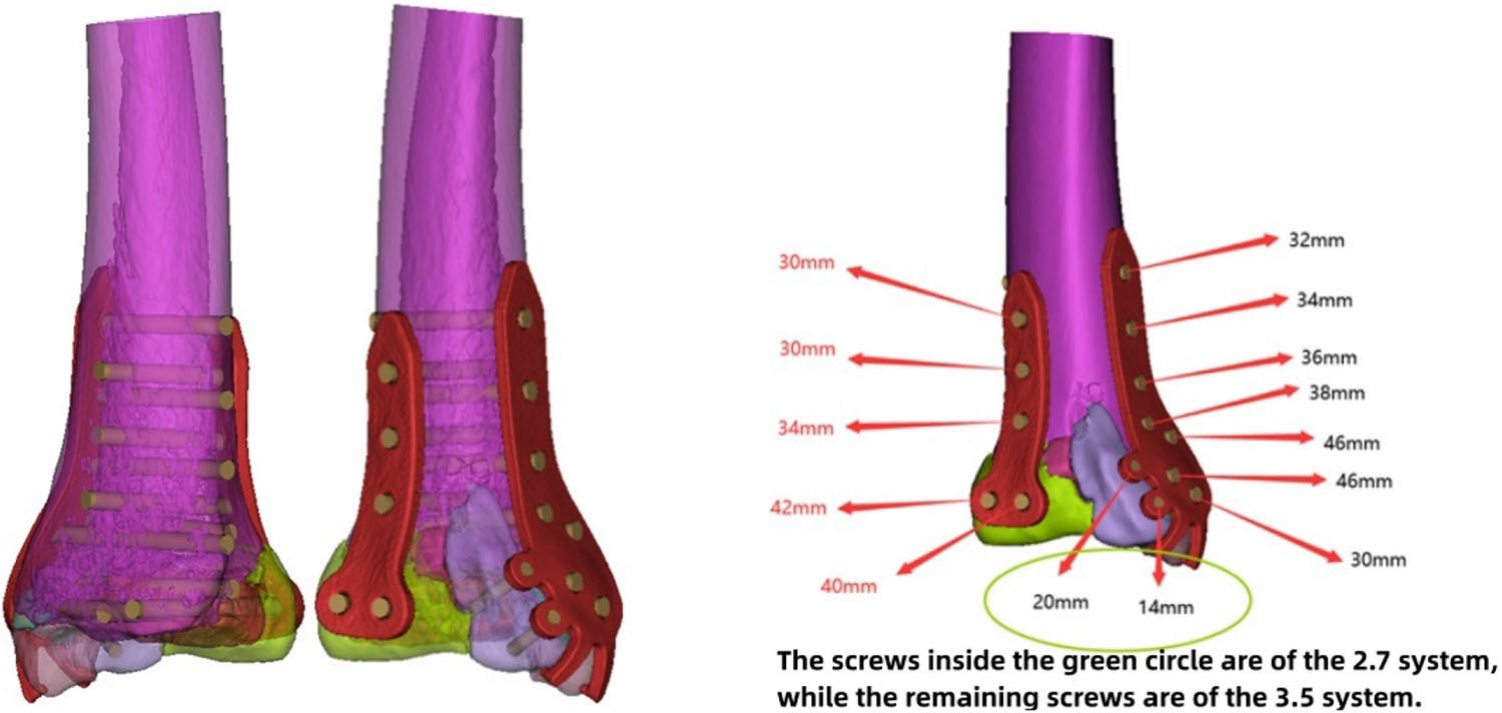
Transparency adjustments are applied to the fracture model to ensure that implanted screws do not penetrate the ankle joint cavity. Measurement of the screw length for fixation and marking of recommended lengths beside the screw holes.

**Figure 4:**
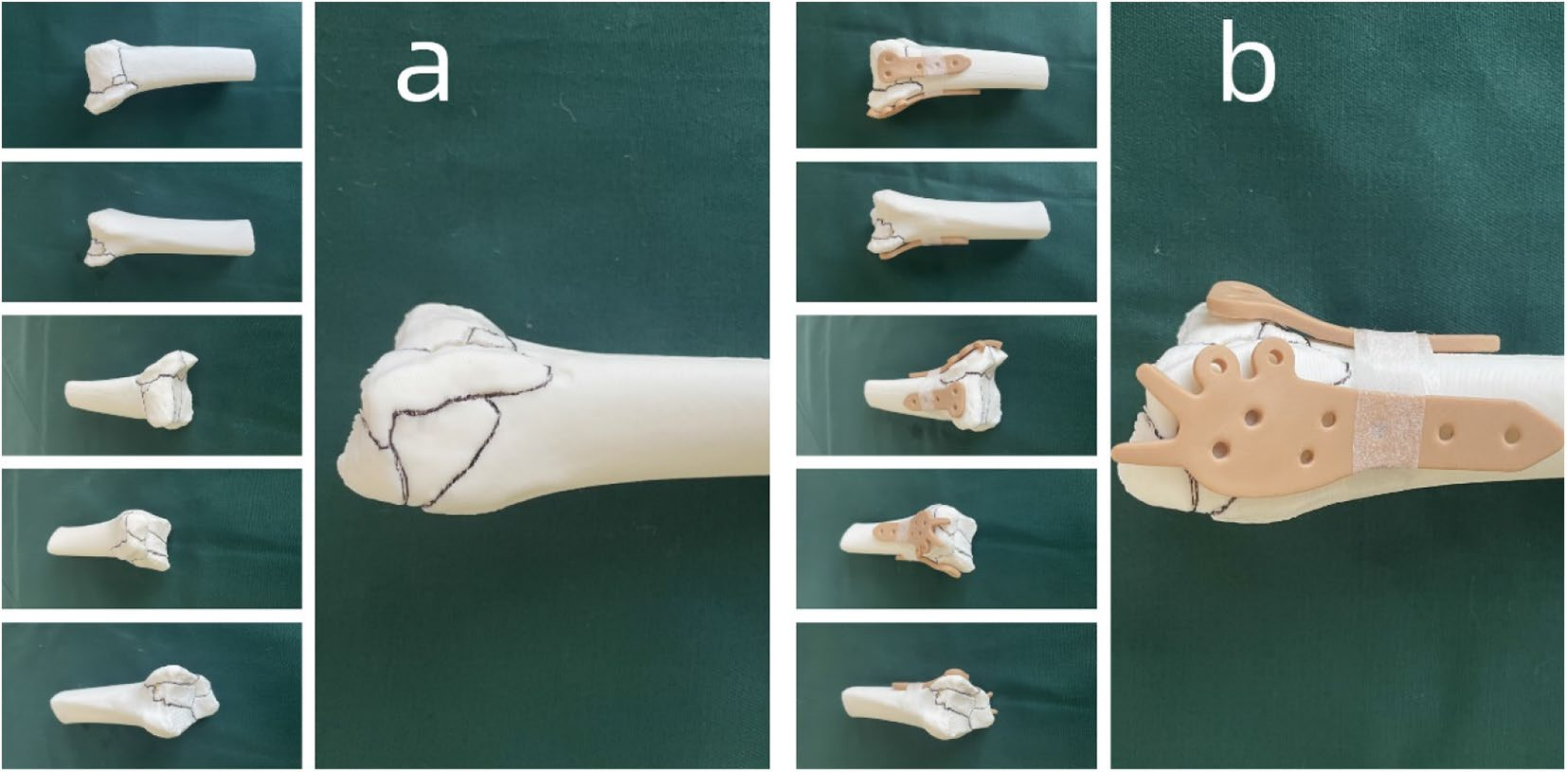
(**a**) 3D printed model after virtual fracture reduction. (**b**) Overlay simulation of the virtually reduced fracture 3D model and the plate model. Properly customized plates and fracture 3D models were disinfected and kept on standby for use during surgery.

Subsequent engineering involved detailed design of the plate using Unigraphics NX software (Siemens PLM Software, USA). The physical model of the plate was printed using FlashPrint 5 software (Fig. 4b). The physical model of the plate was then imported into Mimics software through reverse scanning to confirm the position, length, direction, and diameter of the screws (Fig. 2). The transparency of the fracture model was adjusted to ensure that the implanted screws would not enter the ankle joint cavity, and the recommended length of the screws was marked beside each hole in the plate (Fig. 3). The customized individualized plate was manufactured using pure titanium TA3 as the raw material. The plate underwent detailed inspection and sterilization in the processing plant.

### Surgical procedure and brief overview

All surgeries were performed by the same team of surgeons. The surgical approach utilized common anterior-medial and anterior-lateral approaches to the distal tibia.

#### Individualized plate group (standard surgical procedure)

##### Standard surgery in the lateral malleolus

The patient was positioned in a lateral decubitus floating position. The initial surgery involved the lateral malleolus, with a vertical incision on the lateral aspect exposing the fracture. Typically, fractures of the distal fibula were comminuted. The fracture was reduced, assessed under fluoroscopy, and fixed with an anatomical locking plate and screws for the distal fibula. Ankle joint motion in various directions was performed to assess the strength and stability of the fracture fixation.

##### Subsequent surgery on the posterior malleolus

Separation through the interval behind the peroneal muscles exposed the posterior malleolus. Typically, the posterior malleolus fracture presented as comminuted or segmental fragments. Combining the 3D printed model for fracture reduction (Fig. 4a), the fracture was anatomically reduced, restoring the joint surface. A customized individualized plate and screws were then applied (Fig. 4b), and intraoperative fluoroscopy was used to assess the reduction and fixation of the posterior malleolus fracture comprehensively.

##### Final surgery on the medial malleolus

An arched incision was made at the medial malleolus, exposing the fracture. In selected cases, the medial malleolus fracture was typically comminuted. Utilizing the 3D printed model for fracture reduction (Fig. 4a), a customized individualized plate was applied for fixation (Fig. 4b). Intraoperative fluoroscopy was used to assess the reduction and ensure precise and comprehensive internal fixation.

##### Conventional plate group (standard surgical procedure)

The surgical approach and fixation method for the conventional plate group were the same as those for the individualized plate group, using standard plates and screws for fixation.

### Postoperative management

**Figure 5:**
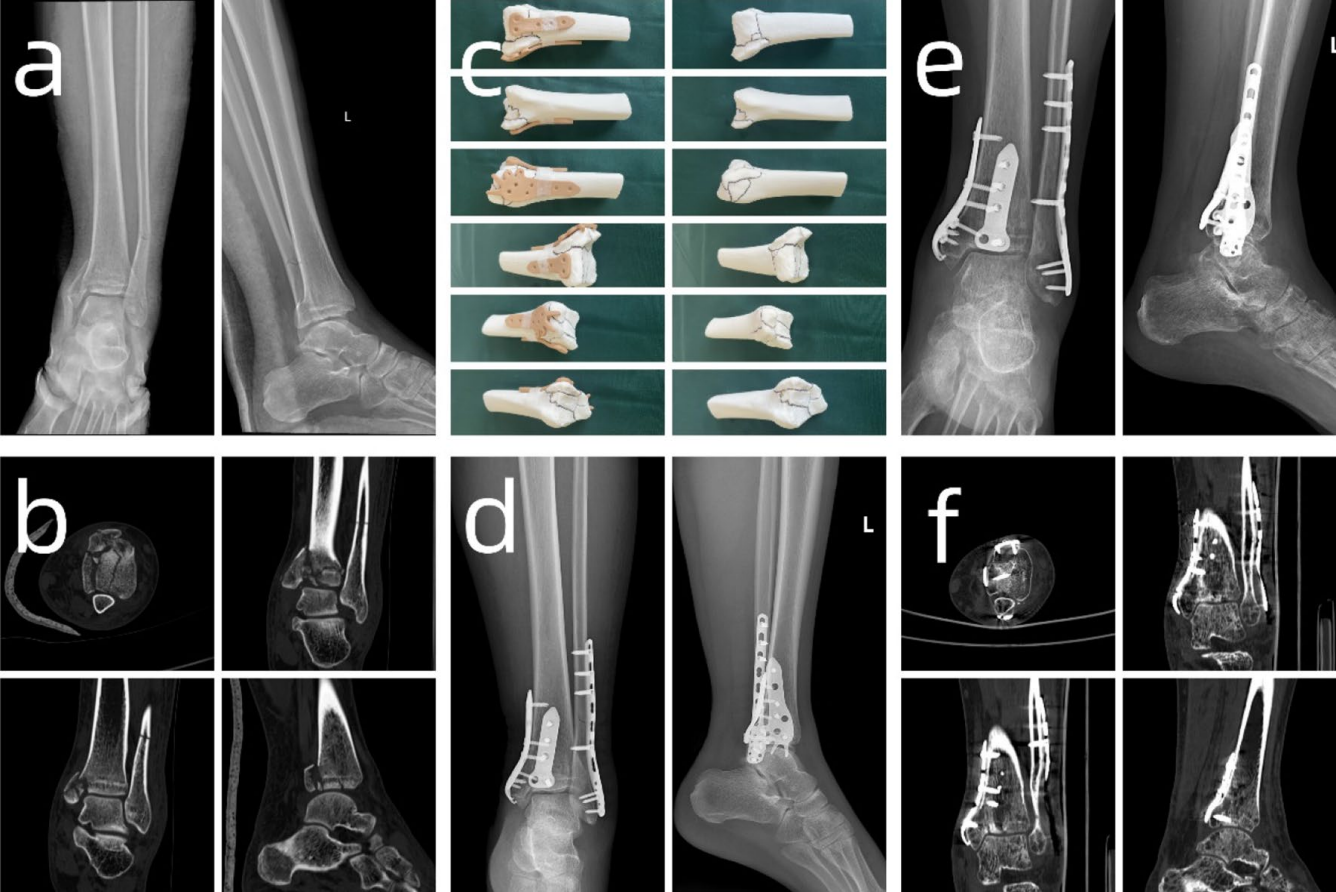
Case data of a 33-year-old patient with complex distal tibia fracture treated with an individualized steel plate system. (**a**) Preoperative ankle joint X-ray (anteroposterior and lateral views). (**b**) Preoperative ankle joint CT scan. (**c**) 3D-printed models of the fracture, plate, and fit simulation. (**d**) Postoperative day 1 ankle joint X-ray (anteroposterior and lateral views). (**e**) 6-month follow-up ankle joint X-ray (anteroposterior and lateral views). (**f**) 6-month follow-up ankle joint CT scan.

Retaining the patient's preoperative X-ray (Fig. 5a), preoperative CT scan (Fig. 5b), preoperative fracture reduction model, and plate fitting model data (Fig. 5c). Performing a postoperative day one reexamination with ankle joint anteroposterior and lateral X-ray images (Fig. 5d) and comparing them to the patient's preoperative radiographic and model data for the assessment of fracture reduction using the Burwell–Charnley radiographic evaluation^13^. Once anesthesia recovery was achieved, patients were instructed to initiate gentle active and passive ankle dorsiflexion-plantarflexion exercises. Additionally, muscle contraction and relaxation exercises for the knee and hip joints were commenced. Around one week postoperatively, partial weight-bearing strength exercises were introduced and gradually intensified. At approximately two weeks post-surgery, patients were guided in crutch walking exercises. Follow-up appointments were scheduled at 1, 2 and 3 months post-surgery for ankle joint anteroposterior and lateral X-ray imaging to evaluate ankle joint recovery and fracture healing. Patients were also provided guidance on postoperative rehabilitation exercises and gradual return to full weight-bearing activities. At 6 and 12 months postoperatively, a discretionary follow-up will include ankle joint anteroposterior and lateral X-ray images (Fig. 5e) and postoperative ankle CT scans (Fig. 5f) to assess the patient's post-fracture recovery.

### Clinical evaluation indicators

Preoperative preparation time refers to the total time from the patient's injury to their entry into the operating room before surgery. Hospitalization time is the duration from the patient's admission to discharge. Surgical time is the total time from the skin incision to the completion of skin closure. Fracture reduction and internal fixation time encompass the time from the beginning of adequate fracture exposure to the completion of internal fixation device fixation. This includes fracture reduction, internal implant adjustments, drilling, screw length measurement, and locking of internal fixation devices. The total length of the surgical incision is measured and recorded after completion of suturing.

All radiological evaluations were conducted by three experienced orthopedic surgeons. On the first day after surgery, ankle joint X-ray images in both anterior–posterior and lateral views were obtained and evaluated for fracture reduction quality using the Burwell–Charnley criteria: fracture reduction quality was categorized as good (displacement < 1–2 mm) or fair (displacement > 2–5 mm).

Follow-up assessments were conducted, and at the 12-month follow-up, ankle joint function recovery was subjectively evaluated using the Mazur ankle function scoring criteria^14^: ankle joint function was classified as excellent/good (Mazur score ≥ 87) or fair/poor (Mazur score < 87).

Complications analyzed in the study included postoperative incision infection, skin necrosis, delayed/non-union of fractures, implant failure, joint function impairment (reduced mobility, pain requiring medication control), heterotopic ossification, and post-traumatic arthritis.

### Statistical analysis

All statistical analyses were performed using SPSS software (version 26.0, USA). Continuous variables are presented as mean ± standard deviation. Categorical variables are presented as numbers or percentages. Fisher's exact test was used to assess categorical variables. The normality of continuous variables was assessed using the Shapiro–Wilk test. For normally distributed continuous variables, independent-sample t-tests were used for analysis. For non-normally distributed continuous variables, the Mann–Whitney U test was employed for analysis. The significance level for all statistical tests was set at p < 0.05.

### Consent to participate

Informed consent was obtained from all individual participants included in the study.

## Results

### Baseline and hospitalization information

A total of 48 patients were included in this study, with 24 in the individualized plate group. Among them, 15 were male and 9 were female, with an age range of 20–62 years and an average age of (42.8 ± 10.1) years, and a BMI of (24.5 ± 3.3). In the conventional plate group, there were 24 patients, including 13 males and 11 females, with an age range of 21–64 years and an average age of (40.7 ± 10.1) years, and a BMI of (25.2 ± 3.2). The baseline characteristics of the two groups were statistically similar (p > 0.05) (Table 1).

**Table 1:** Patient baseline and hospitalization information.

Parameters	Individualized plate group (n = 24)	Conventional plate group (n = 24)	p value
Age (years)	42.8 ± 10.1	40.7 ± 10.1	0.469
Gender			0.558
Male	15	13	
Female	9	11	
BMI (kg/m^2^)	24.5 ± 3.3	25.2 ± 3.2	0.359
Preoperative time (h)	135.0 ± 6.0	98.3 ± 7.5	< 0.001
Hospital stay (days)	10.5 ± 0.8	9.0 ± 0.7	< 0.001
3D printing time (min)	640.6 ± 40.7		
Custom plate time (h)	47.8 ± 2.3		

The preparation time before surgery and hospitalization time in the individualized plate group were slightly longer than those in the conventional plate group ((135.0 ± 6.0) h vs (98.3 ± 7.5) h, p < 0.001; (10.5 ± 0.8) days vs (9.0 ± 0.7) days, p < 0.001), with significant differences (p < 0.001). This difference may be attributed to the impact of 3D printing time and the customization time of individualized plates in the individualized plate group ((640.6 ± 40.7) min; (47.8 ± 2.3) h) (Table 1).

### Surgical information

In the individualized plate group, the surgical time, fracture reduction and internal fixation time were significantly shorter compared to the conventional plate group ((72.7 ± 2.8) min vs (95.3 ± 2.8) min, p < 0.001; (37.2 ± 3.0) min vs (60.5 ± 3.1) min, p < 0.001). The individualized plate group also had a reduced number of intraoperative fluoroscopic examinations (5 ± 0.8 vs 8 ± 0.8, p < 0.001). Moreover, the total length of surgical incisions in the individualized plate group was shorter ((11.5 ± 0.6) cm vs (13.4 ± 0.7) cm, p < 0.001). All differences were statistically significant (p < 0.05) (Table 2).

**Table 2:** Patient surgical, postoperative and follow-up information.

Parameters	Individualized plate group (n = 24)	Conventional plate group (n = 24)	p value
Surgery time (min)	72.7 ± 2.8	95.3 ± 2.8	< 0.001
Fracture reduction and internal fixation time (min)	37.2 ± 3.0	60.5 ± 3.1	< 0.001
Intraoperative fluoroscopy (frequency)	5 ± 0.8	8 ± 0.8	< 0.001
Incision length (cm)	11.5 ± 0.6	13.4 ± 0.7	< 0.001
Fracture healing time (weeks)	11.0 ± 1.1	11.5 ± 1.2	0.126
Follow-up time (months)	15.7 ± 2.8	15.0 ± 2.6	0.433
Fracture reduction degree n (%)			0.439
Excellent reduction (< 1 mm)	21 (87.5)	19 (79.2)	
Fair reduction (> 2-5 mm)	3 (12.5)	5 (20.8)	
Mazur score n (%)			0.033
Excellent/good group (≥ 87 points)	22 (91.7)	16 (66.7)	
Fair/poor group (< 87 points)	2 (8.3)	8 (33.3)	
Complications n (%)			0.22
Present	2 (8.3)	5 (20.8)	
Absent	22 (91.7)	19 (79.2)	

### Postoperative and follow-up information

The time to fracture healing was similar between the individualized plate group and the conventional plate group ((11.0 ± 1.1) weeks vs (11.5 ± 1.2) weeks, p > 0.05). The follow-up duration was comparable between the two groups (individualized plate group: (15.7 ± 2.8) months, conventional plate group: (15.0 ± 2.6) months, p > 0.05), and there were no lost-to-follow-up patients. The rate of successful fracture reduction was higher in the individualized plate group compared to the conventional plate group (87.5% vs 79.2%, p > 0.05), but the difference was not statistically significant. Moreover, the ankle joint functional scores in the individualized plate group were superior to those in the conventional plate group (excellent/good ratio: 91.7% vs 66.7%, χ^2^ = 4.5, p < 0.05) (Table 2).

There were two cases of complications in the individualized plate group, including one case of postoperative wound infection and one case of ankle joint functional impairment. In the conventional plate group, there were five cases of complications, including two cases of postoperative wound infection and three cases of ankle joint functional impairment. Although the complication rate in the individualized plate group was lower than that in the conventional plate group (8.3% vs 20.8%, p = 0.22), the difference was not statistically significant (Table 2).

## Discussion

Complex distal intra-articular fractures of the trimalleolar ankle involve fractures within the joint, with the ankle joint bearing weight. During surgery, achieving anatomical reduction and robust fixation are key factors for patients to achieve a good recovery. In most patients, there is swelling in the affected limb, and the condition of the soft tissues is often poor. Performing surgery hastily during the swelling stage may worsen the swelling, affect blood circulation at the site, and increase the risk of muscle soft tissue necrosis and infection^15,16^. By utilizing 3D printing models for comparing fracture reductions and then applying customized plates for effective fixation, patients with ankle joint fractures can receive more precise and personalized treatment. The proficiency of surgical procedures and the experience of the surgeon do influence surgical outcomes. However, due to significant individual variations in these fractures, robust preoperative planning, simulation exercises, and the use of customized plates and tailored surgical approaches contribute to effectively enhancing surgical quality and achieving optimal clinical treatment results^5,17,18^.

This study indicates that the personalized plate group had slightly longer preoperative preparation and hospitalization times compared to the conventional plate group, and the difference was statistically significant. This may be influenced by the preoperative printing of 3D models and customized plates. With the development of printing and customization technologies, researchers believe that the waiting time in this regard will likely decrease in the future^19^. In the study, the combination of 3D printing technology and personalized customized plates, when compared to the conventional plate group, showed significantly reduced surgical time, fracture reduction, and internal fixation operation time. There were fewer intraoperative fluoroscopy sessions, and the total length of the surgical incision was shortened. According to the follow-up, the Mazur ankle joint function recovery level was satisfactory, and the differences were statistically significant (p < 0.05). The personalized plate group showed a higher rate of good fracture reduction according to the Burwell–Charnley score, but the difference was not statistically significant. The complication rate in the personalized plate group was lower than that in the conventional plate group (2/24, 8.3% vs. 5/24, 20.8%, p = 0.22), but the difference was not statistically significant. It is noteworthy that the complications in the personalized plate group were not related to 3D printing technology and personalized customized plates.

In recent years, the application of 3D printing technology in orthopedics has become increasingly widespread. By 3D printing fracture models, patients and physicians can obtain more intuitive and three-dimensional fracture information, allowing for more comprehensive preoperative planning and simulation. Related studies also indicate that 3D printing technology-assisted fracture surgery, compared to traditional surgery, results in better surgical outcomes and higher satisfaction among both doctors and patients^20–22^. However, current 3D printing technology is primarily used for pre-shaping conventional plates, which can affect their strength and may lead to incomplete and unstable fixation of fracture fragments. In this study, our team customized plates based on virtually reconstructed fracture models, considering factors such as strength, fit, comprehensive fixation, and stability. Yoshii et al.^23^ confirmed the significant benefits of pre-determining the position and type of plates and screws for distal radius fractures. Shuang et al.^12^ compared the efficacy of traditional plates with custom personalized plates in the treatment of distal humeral fractures, with personalized plates achieving more satisfactory postoperative joint function recovery. Yang et al.^11^ demonstrated the effectiveness of 3D-printed customized personalized plates in orthopedic surgery for fractures in the head and neck, simplifying surgical procedures and achieving more satisfactory outcomes through highly accurate planning. Personalized customized plates are not affected by individual differences in fractures and can be tailored to each patient's specific needs^7^. At the same time, comprehensive, strong, and stable internal fixation provides conditions for patients to start early exercises. Jansen et al.'s research confirmed that active controlled movement after early ankle fracture surgery leads to better clinical and functional outcomes^24^. Smeeing et al.^25^ demonstrated that early active movement and weight-bearing are safe choices that promote a quicker return to work and daily activities. With the further development of technology and increasing demands for orthopedic surgery and postoperative recovery, the use of 3D printing technology and personalized customization in orthopedics has become a reality^26^.

However, the combined use of 3D printing technology and personalized custom-made plates still faces two significant "stubborn" challenges: firstly, the cost of custom-made plates is high, with substantial expenses associated with 3D printing equipment, printing materials, and material requirements (including biocompatibility and degradability)^27,28^. Secondly, although 3D printing is a rapid prototyping technique, current research and circumstances indicate that both printing and customization processes still require a significant amount of time^29^.

The limitations of this study include the small sample size of only 48 cases and the lack of long-term follow-up of 3–5 years. These factors could introduce some bias into the results. It is hoped that future high-quality studies with larger and more diverse samples will provide more comprehensive and accurate conclusions.

## Conclusion

This study utilized 3D printing technology in conjunction with personalized customized plates for individualized medical treatment, resulting in savings in surgical time for complex comminuted trimalleolar ankle fractures. There was a reduction in the time required for fracture reduction and internal fixation, a decrease in the number of intraoperative fluoroscopy sessions, and a shorter total length of the surgical incision. This approach improved the rate of successful fracture reduction, and through long-term follow-up, it demonstrated more satisfactory outcomes in terms of ankle joint functional recovery.

## Data Availability

The datasets generated and/or analysed during the current study are not publicly available due to limitations of ethical approval involving the patient data and anonymity but are available from the corresponding author on reasonable request.
